# Case of Hæmato-Scrofulous Tumour of Enormous Size, with the Appearances on Dissection

**Published:** 1817-05

**Authors:** 


### *Case of Hæmato-Scrofulous Tumour of enormous Size, with the Appearances on Dissection.*

(Communicated by Dr. JOHNSON.)

MISS B. ætat. 16, tall, slender, and of the strumous appearance, experienced a fall on the left knee, about seven months previously to Christmas, 1815. Ever after that period she felt pain in that joint. In December, a small swelling was perceived on the inner condyle, which gradually increasing, spread round the upper part of the joint; and by the 22d of May, 1816, it had attained the size of a very large man's head, and extended about half way up the thigh. It was uniformly hard, and seemed equally to embrace the femur and upper part of the joint. The colour was not altered, but large blue veins were then apparent on its surface.

By the beginning of August, 1816, it had attained very rapidly a much greater size, and measured 26 inches in circumference. It now projected much more on the inner than on the outer side of the thigh, and was softer there than formerly. It afforded some obscure indications of a contained fluid. All remedial measures had proved unavailing in arresting the progress of the tumour; the poor girl's strength was fast declining, and hectic fever was beginning to develope itself. Much diversity of opinion existed relative to the nature of the disease; but it was universally agreed that nothing but an operation could offer a prospect of life.

### *Operation, 13th August,* 1816.

The first incision (slantingly longitudinal between two large veins) half, or three quarters of an inch in depth, and five or six inches in length, disclosed nothing but unhealthy or gelatinous-looking cellular and adipose substance. The next incision went through the fascia, when a considerable quantity of blood and serous fluid boiled out from the turgid vessels of the tumour, a tourniquet being now moderately tightened on the thigh, near the groin. The deeper incisions developed a mass of disorganization, of various textures and appearances; some parts resembling cerebrum; some condensed cellular substance; some ligament; some jelly; some marrow; and some diseased fat. Throughout the greater extent of the tumour were interspersed very many cells, bags, or cavities, of extremely irregular shapes, filled with fluids, principally serum and coagulable lymph; but, in some places, coagulated blood, apparently recently extravasated; and in several of these sacs was found afterwards, on more minute dissection, ill-conditioned purulent matter, very thick in consistence. The artery was traced (after the limb was removed) from the triceps, through the ham, to the leg, but was perfectly pervious and sound. The condyles of the femur were amazingly enlarged and diseased. They were spread to full six inches in breadth; and in some cases so soft, that the scalpel went through them, in all directions, without meeting more resistance than was offered by the circumvesting integuments. The cartilages of the femur were not abraded; but they readily stripped off the condyles of the bone, which were so rotten and soft underneath, that the finger could be pushed into them. The semilunar cartilages were apparently unaffected; but the crucial ligaments were rotten. The head of the tibia, especially on the inner side, was scabrous, spongy, and carious. The cartilage was abraded off the internal edge of the patella, and the bone itself was scabrous and diseased in that place. The articulating cartilages of the tibia were every where sound. The body of the femur was diseased and enlarged, for the space of eight inches from the joint. Towards the condyles it was soft and carious; but upwards, towards the middle of the bone, it was enlarged, and pretty hard. Several calcareous depositions were found scattered through different parts of this tumour.

Two portions of the cerebroid substance were put, one into sulphuric acid, and the other into water. The *former* was soon dissolved, and formed a black, oily, homogeneous liquid; the *latter* rendered the water turbid, but did not dissolve.

Mr. Burns mentions his having seen white swelling and fungus hæmatodes united in the same tumour; and the present case appears to confirm this assertion. Various were the opinions, both before and after the removal of the limb, respecting the nature of the disease. Several able men, who had not seen the progress of the tumour, pronounced it aneurism, even on the day of the operation.

The thigh was removed at the little trochanter, and half a minute's dissection would have liberated the head of the bone from the acetabulum. Would not this be a better plan of amputating at the hip joint, after all that has been said on the subject?—The girl recovered; but she nearly expired on the table.

